# Supplementary material for: Soluble αβ-tubulins reversibly sequester TTC5 to regulate tubulin mRNA decay
Source: Nat Commun. 2024 Nov 17;15:9963. doi: 10.1038/s41467-024-54036-0 (PMC11570694; doi:10.1038/s41467-024-54036-0)
Supplement: Supplementary file 1 — Supplementary Information [file 41467_2024_54036_MOESM1_ESM.pdf]

Supplementary tables, figures, figure titles and legends for manuscript:

Soluble αβ-tubulins reversibly sequester TTC5 to regulate tubulin mRNA decay

Alina Batiuk[1], Markus Höpfler[2], Ana C. Almeida[1], Deryn Teoh En-Jie[3], Oscar Vadas[4], Evangelia Vartholomaiou[1], Ramanujan S. Hegde[2], Zhewang Lin[3*], Ivana Gasic[1*]

[1] Department of Molecular and Cellular Biology, University of Geneva, Geneva, Switzerland

[2] Medical Research Council Laboratory of Molecular Biology, Cambridge CB2 0QH, United Kingdom

[3] Department of Biological Sciences, National University of Singapore, Singapore

[4] Proteins, peptides and RNA to protein core facility, Faculty of Medicine, University of Geneva, Switzerland

[*] *Correspondence to ivana.gasic@unige.ch and zlin@nus.edu.sg*

Table of contents:

1. **Supplementary Figure 1 and figure legend**
2. **Supplementary Figure 2 and figure legend**
3. **Supplementary Figure 3 and figure legend**
4. **Supplementary Figure 4 and figure legend**
5. **Supplementary Table 1**
6. **Supplementary Table 2**

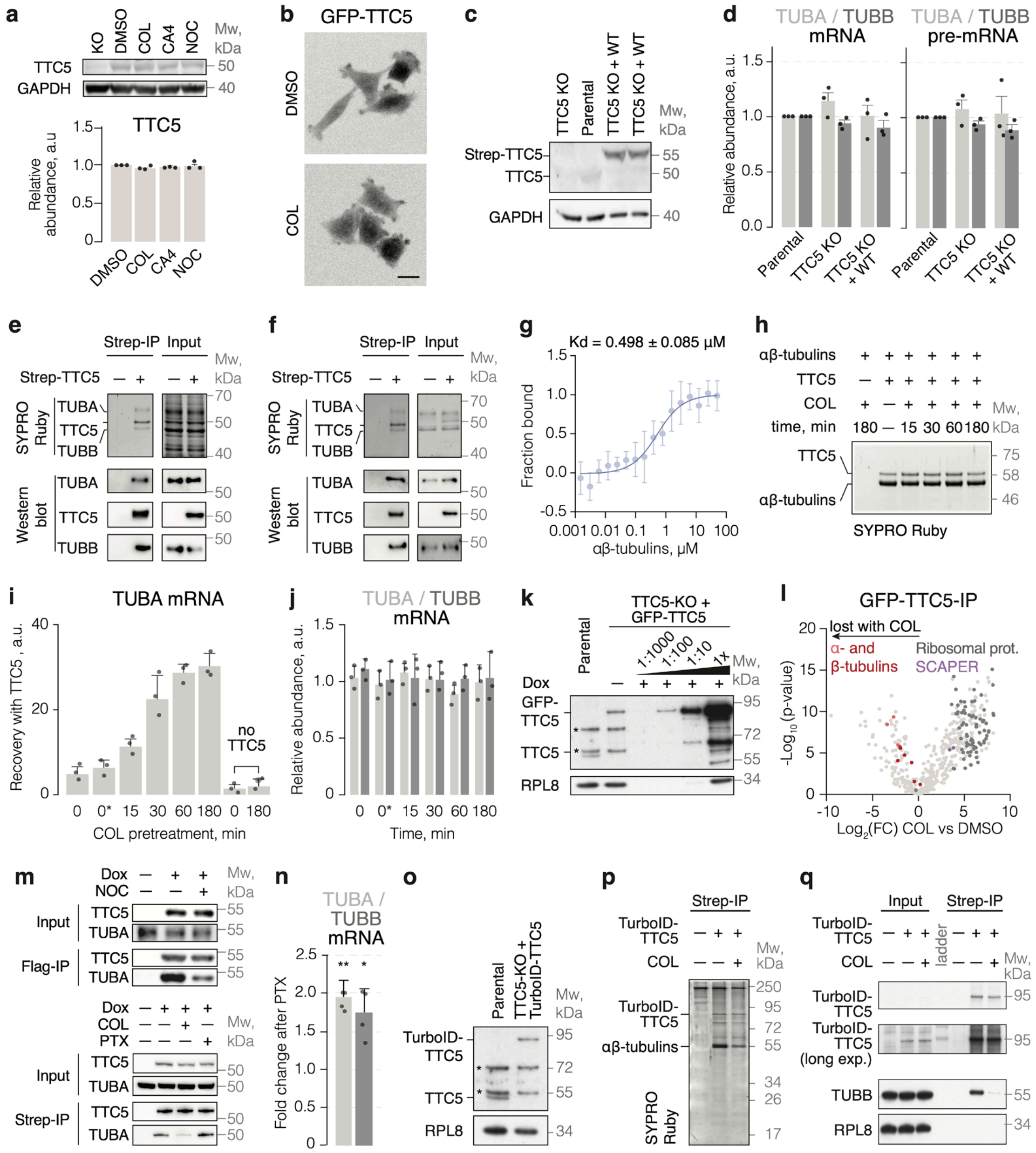

Figure S1. Soluble αβ-tubulins reversibly repress TTC5 to regulate its activity (related to Figure 1). a Total cell lysates from HeLa TTC5 knockout (KO), and HeLa parental cells treated with DMSO vehicle, or 1 µM colchicine (COL), combretastatin A4 (CA4) or nocodazole (NOC) for 5 hours. Proteins were separated by SDS-PAGE and visualized by western blot (upper panel). TTC5 levels across the indicated conditions were normalized to GAPDH and presented as mean ± SD from three independent replicates (bottom panel). **b** Representative images of HeLa TTC5 knockout cells re-expressing GFP-TTC5, after treatment with DMSO vehicle control or 1 µM colchicine (COL) for 5 hours. Scale bar = 10 µm. **c** Western blot demonstrating the endogenous

and Strep-TTC5 expression in total cell lysates across the indicated cell lines. **d** Relative α- and β-tubulin mRNA and pre-mRNA levels in HeLa parental, TTC5 knockout, and Strep-TTC5^{WT} rescue cell lines, normalized to a housekeeping transcript and the parental cell line. Data show the mean ± SD from three independent experiments. No statistically significant differences were detected in unpaired, two-tailed Student's t tests for each of the indicated cell lines with the parental cell line as reference. **e** HEK 293 TTC5 knockout cells transfected with a plasmid encoding Strep-TTC5 under a doxycycline-inducible promoter were lysed and Strep-TTC5 was affinity purified via the Twin Strep-tag. Total lysate input and the coimmunoprecipitated interactors (Strep-IP) were separated using SDS-PAGE that resulted in a high-resolution separation of α- and β-tubulin monomers and visualized by SYPRO Ruby staining or western blot. **f** Recombinant Strep-TTC5 was incubated with porcine brain tubulin, then affinity purified via the Strep-tag. Pulled down proteins were separated by SDS-PAGE and visualized using SYPRO Ruby total protein stain and western blot, showing a stoichiometric ratio of α- and β-tubulins pulled down with Strep-TTC5. **g** Dissociation constant of porcine tubulin and TTC5 determined by microscale thermophoresis. **h** Recombinant Strep-TTC5 was incubated with porcine brain tubulin in the absence or presence of 1 μM colchicine at 37 °C and the indicated time durations, then affinity purified via the Strep-tag. Pulled down proteins were separated by SDS-PAGE and visualized using western blot, showing that binding of TTC5 to purified porcine brain tubulin is not affected by prolonged incubation with colchicine *in vitro*. **i** Products recovered from cell lysates by binding to recombinant TTC5 shown in Figure 1e were analyzed for α-tubulin mRNA by quantitative RT-PCR (mean ± SD from three independent replicates). **j** Total α- and β-tubulin mRNA levels from TTC5 KO cells used in Figure 1e and 1f (mean ± SD from three independent replicates). No statistically significant differences were detected in Student's t-test. **k** Western blot analysis of total cell lysates of the parental and HEK293 TTC5 knockout cells re-expressing GFP-TTC5. Asterisks indicate unspecific bands. **l** Total cell lysates of HEK293 TTC5 knockout cells re-expressing doxycycline-inducible GFP-TTC5 and treated either with DMSO vehicle control or 10 μM colchicine for 1–3 hours were immunoprecipitated using the GFP-tag. TTC5 interactome was subsequently analyzed using label-free quantitative mass spectrometry. Data are presented as Log_2 fold change (Log_2(FC)) in colchicine versus DMSO treated samples. Highlighted are the TTC5 interaction partners specifically lost (tubulins, red) or gained (ribosomal proteins, dark gray, and SCAPER, purple) upon colchicine treatment. Processed data are provided in Supplementary Data 2. **m** HeLa TTC5

knockout cells re-expressing Flag-TTC5 under a doxycycline-inducible promoter were treated with DMSO or 1 μM nocodazole (NOC) for 3 hours. HEK 293TTC5 knockout cells re-expressing strep-TTC5 under a doxycycline-inducible promoter were treated with DMSO or 10 μM colchicine (COL) or 3 μg/mL Paclitaxel (PTX) for 3 hours. Cells were then lysed and TTC5 was affinity purified via the Flag-tag or Strep-tag respectively. Coimmunoprecipitated interactors were separated using SDS-PAGE and visualized using western blot. **n** Autoregulation assay in HeLa cells showing the mean ± SD mRNA levels after paclitaxel (PTX) treatment from three independent experiments. Single asterisk indicates $p < 0.05$, and double $p < 0.01$ in unpaired, two-tailed Student's t tests with the DMSO-treated sample as reference. **o** Western blot analysis of total cell lysates of the parental and HEK293 TTC5 knockout cells re-expressing doxycycline-inducible TurboID-TTC5 demonstrating near-endogenous levels of TurboID-TTC5 when no doxycycline is added. Asterisks indicate unspecific bands. **p-q** HEK 293 TTC5 knockout cells re-expressing TTC5 N-terminally fused to TurboID biotin ligase, under doxycycline-inducible promoter, were treated with DMSO vehicle or 10 μM colchicine and incubated in the presence of 50 μM biotin for 2.5 hours. Biotynylated proteins were affinity purified, separated by SDS-PAGE and visualized using SYPRO Ruby stain (p) or western blot (q).

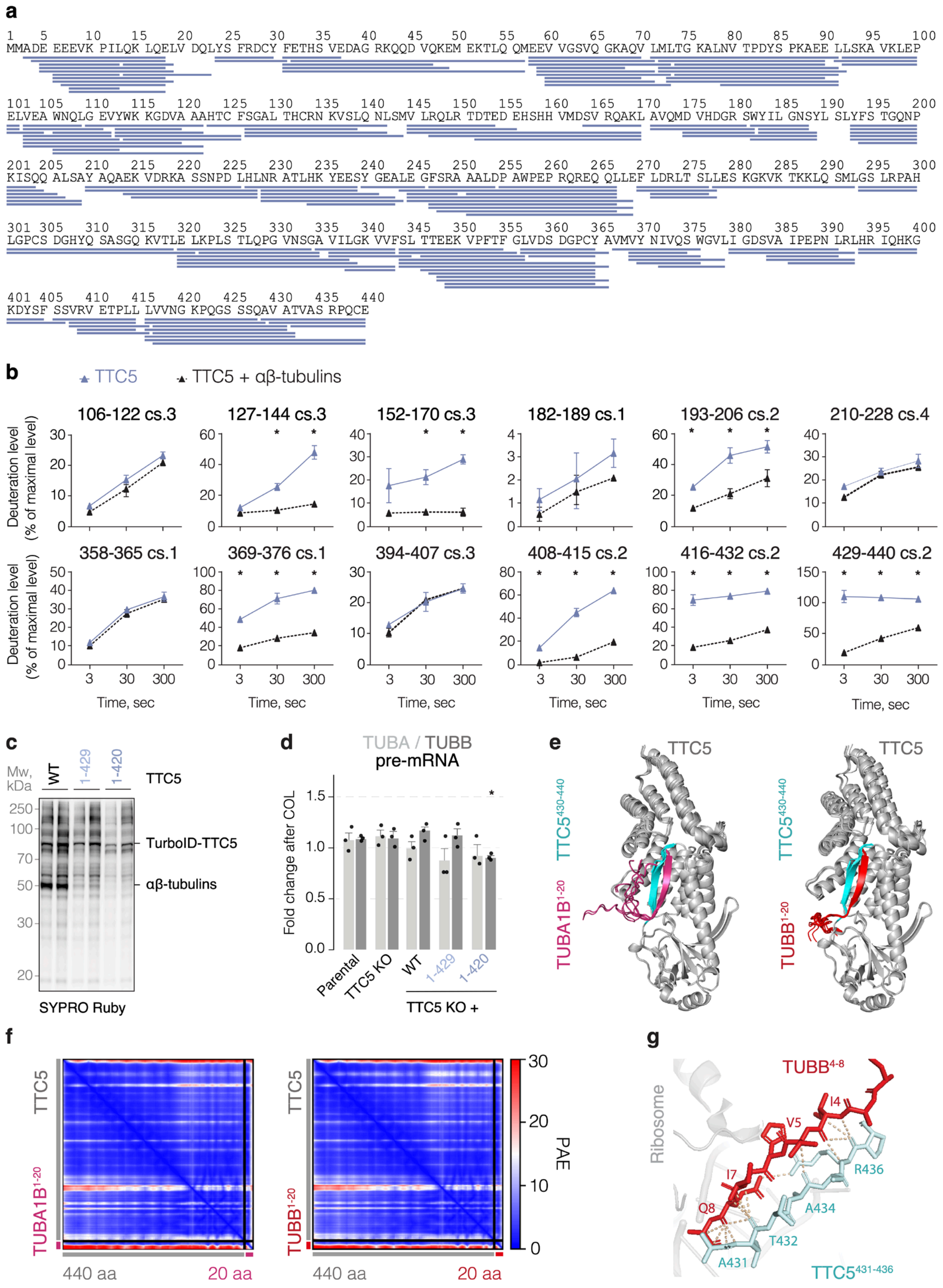

Figure S2. C-terminal tail mediates interaction with mature and nascent tubulin (related to Figure 2). a Peptide coverage for recombinant Strep-TTC5 related to the HDX-MS analysis presented in Figure 2a-d. **b** Deuteration profiles of a selection of TTC5 peptides upon incubation

in deuterated water for the indicated time durations (mean ± SD from three independent replicates). **c** HeLa TTC5 knockout cells re-expressing TTC5 N-terminally fused with TurboID biotin ligase under doxycycline-inducible promoter were incubated in the presence of 50 µM biotin for 2.5 hours. Biotinylated proteins were separated by SDS-PAGE and visualized using western blot. Data from two biological replicates are shown. **d** Autoregulation assay with HeLa parental, TTC5 knockout, and the indicated Flag-TTC5 rescue cell lines. Data show the mean ± SD of pre-mRNA levels after colchicine treatment, from at least three independent experiments. Single asterisk indicates $p < 0.05$, in unpaired, two-tailed Student's t-tests for each of the indicated cell lines with the DMSO-treated sample as reference. **e** Overlay of ten AlphaFold2 multimer-predicted models of TTC5 (gray) bound to nascent α- (pink, left) or β-tubulin (red, right). C-terminal tail of TTC5 (cyan) is predicted to form an anti-parallel beta sheet with nascent α- (pink) and β-tubulins (red). **f** Predicted aligned error (PAE) for the top ranked models in panel e. **g** Close-up view of the C-terminal tail of TTC5 (cyan) and nascent β-tubulin (red) forming a beta sheet. Yellow dashed lines indicate possible interactions between TTC5 (cyan) and nascent β-tubulin (red).

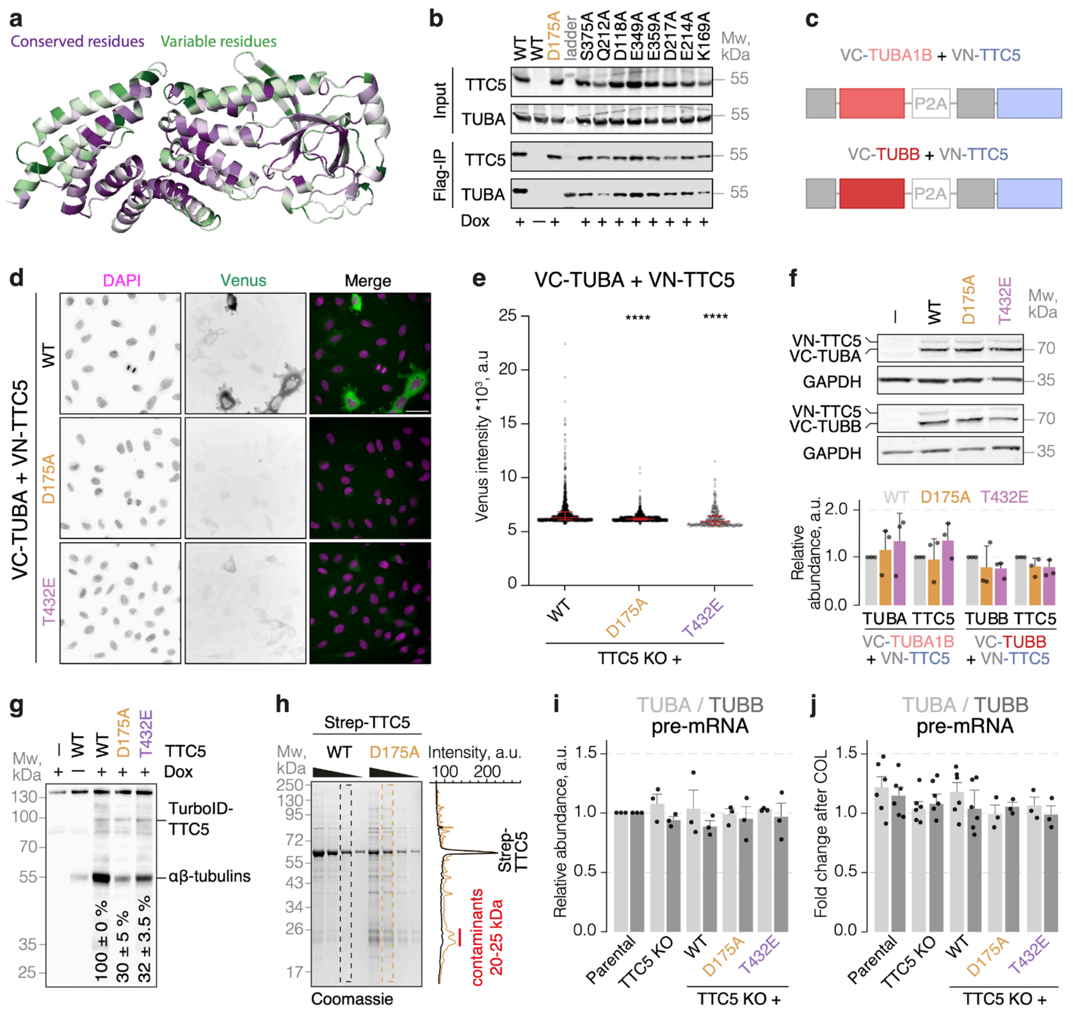

Figure S3. Loss of binding to αβ-tubulins constitutively activates TTC5 (related to Figure 3). **a** Multiple sequence alignment of TTC5 protein mapped onto the AlphaFold2-predicted structure of the human TTC5. Least conserved amino acids are shown in green, and most conserved are shown in purple. **b** Indicated Flag-TTC5 constructs were expressed in HeLa TTC5 knockout cells under a doxycycline-inducible promoter and affinity purified via the Flag-tag. Co-immunoprecipitated interactors were separated using SDS-PAGE and visualized using western blot. **c** Schematic representation of the BiFC constructs. **d** Representative live-cell images of the indicated VN-TTC5 and VC-TUBA constructs expressed in HeLa TTC5 knockout cells. Scale bar = 20 µm. **e** Fluorescence intensity of Venus in cell lines expressing the indicated BiFC constructs. Dots represent measurements from individual cells, red line the median fluorescence intensity, and error bars interquartile range. Quadruple asterisks indicate $p < 0.0001$ in two-sided Mann-Whitney test for each of the indicated BiFC constructs with the BiFC constructs based on TTC5^{WT} as

reference. **f** Total protein analysis by western blot for the indicated BiFC constructs in the cell lines used in panels d and e, and in Figure 3d and 3e. Graph shows relative abundance of BiFC constructs normalized to loading control and $TTC5^{WT}$ (mean ± SD from three independent experiments). No statistically significant differences were detected using unpaired, two-tailed Student's t-test. **g** HeLa TTC5 knockout cells re-expressing TTC5 N-terminally fused with TurboID biotin ligase under doxycycline-inducible promoter were incubated in the presence of 50 μM biotin for 15 minutes. Biotinylated proteins were separated by SDS-PAGE and visualized using western blot. Numbers indicate remaining tubulin labeling (mean ± SD) normalized to the level of the relevant TurboID-TTC5 self-labeling and relative to tubulin biotinylation in cells expressing TurboID-$TTC5^{WT}$. Experiments were done in four biological replicates. **h** Coomassie stained gel (left panel) of the indicated recombinant Strep-TTC5 proteins. Two-fold serial dilutions were loaded for each protein. Note that low molecular weight contaminants are observed in the $TTC5^{D175A}$ sample as indicated by the line intensity profiles (right panel) recorded from the lanes highlighted by dashed rectangles. **i** Total pre-mRNA levels for α- and β-tubulin in the indicated HeLa cell lines. All the data were normalized to a reference transcript and the parental cell line (mean ± SD from three independent experiments). No statistically significant differences were detected using unpaired, two-tailed Student's t-test. **j** Autoregulation assay with HeLa parental, TTC5 knockout, and the indicated Strep-TTC5 rescue cell lines. Data show pre-mRNA levels for α- and β-tubulin normalized to a reference transcript and the DMSO vehicle-treated sample for each tested cell line (mean ± SD from at least three independent experiments). No statistically significant differences were detected using unpaired, two-tailed Student's t-test.

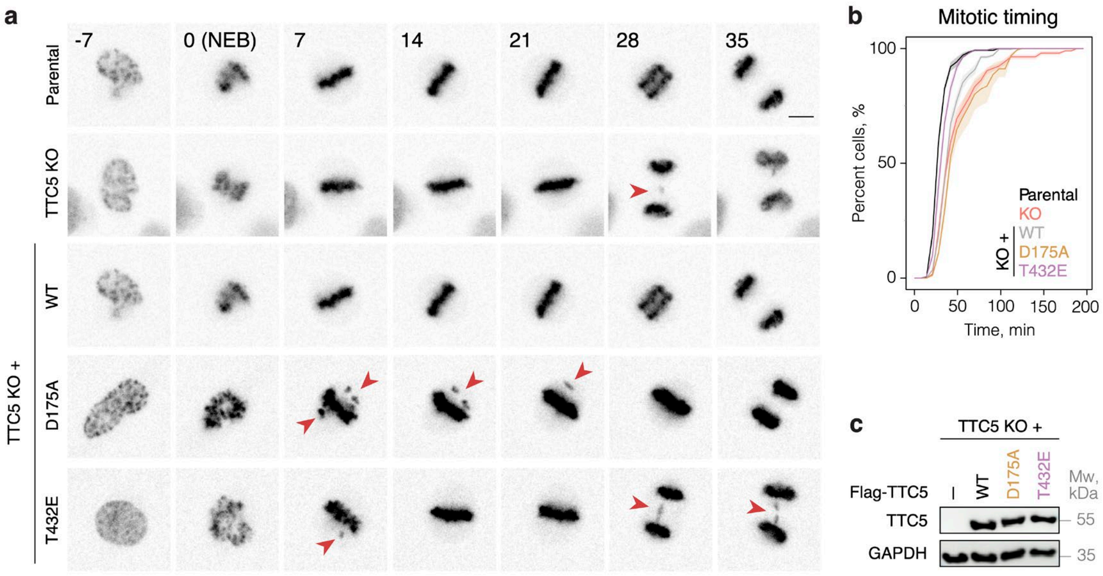

Figure S4. Loss of TTC5 repression by αβ-tubulins impairs mitotic fidelity (related to Figure 4). a Examples from live-cell imaging of HeLa parental, TTC5 knockout, and the indicated Flag-TTC5 rescue cell lines going through mitosis. DNA was visualized using SiR-DNA dye and maximum intensity projections of 3D volumes are shown. Frames were aligned to nuclear envelope breakdown (NEB, t = 0). Misaligned chromosomes in metaphase and segregation errors in anaphase are highlighted with red arrow heads. Scale bar = 10 µm. **b** Mitotic timing (time spent from NEB to anaphase onset) in HeLa cells of the indicated genotypes. Data are presented as cumulative frequencies ± SEM, from at least three independent replicates and more than 100 analyzed cells per genotype. **c** Total protein expression levels analysis by western blot for Flag-TTC5 constructs across the indicated cell lines used in panel a and in Figure 4.

Table S1: Primers used in RT-qPCR experiments

Target gene	Primer	Sequence
PGK1	mRNA_forward	5’ - CCGCTTTCATGTGGAGGAAGAAG - 3’
	mRNA_reverse	5’ - CTCTGTGAGCAGTGCCAAAAGC - 3’
EEF1A1	mRNA_forward	5’ - GATGGCAATGCCAGTGGAACCA - 3’
	mRNA_reverse	5’ - GAGAACACCAGTCTCCACTCGG - 3’
TUBA4A	mRNA_forward	5’ - GGCAAGGAGATCATTGACCCAG - 3’
	mRNA_reverse	5’ - CATCAGGAGTGAGGTGAAGCCA - 3’
	pre-mRNA_forward	5’ - CTTCTTCTGTGAAACTGGTGCTG - 3’
	pre-mRNA_reverse	5’ - TACGGCTAGGAATTTTCAGGGC - 3’
TUBB	mRNA_forward	5’ - GAAGCCACAGGTGGCAAATA - 3’
	mRNA_reverse	5’ - CGTACCACATCCAGGACAGA - 3’
	pre-mRNA_forward	5’ - TCTGTTCGCTCAGGTCCTTT - 3’
	pre-mRNA_reverse	5’ - CCCCTACTGCCCCATAATTT - 3’
GAPDH	pre-mRNA_forward	5’ - GGGAGGTAGAGGGGTGATGT - 3’
	pre-mRNA_reverse	5’ - GAGGCAGGGATGATGTTCTG - 3’
RPL19	pre-mRNA_forward	5’ - TCCGAGAGGTGAAGGCATAG - 3’
	pre-mRNA_reverse	5’ - GCCTCTTCTGAAGCCTGAGC - 3’
RPLP1	mRNA_forward	5 ’- CTCACTTCATCCGGCGACTAG - 3’
	mRNA_reverse	5’ - GCAGAATGAGGGCCGAGTAG - 3’

Table S2: HDX experimental details

HDX experimental details		
Dataset :	State 1	State 2
Description :	TTC5	*TTC5_abTubulin*
Reaction volume :	50 ul	50 ul
% D2O in the reaction :	89.0%	89.0%
temperature :	0 °C	0 °C
D2O incubation times (sec) :	3s, 30s, 300s	3s, 30s, 300s,
Control sample :	Non-deuterated (ND) TTC5	
Quench buffer :	4M Gdn-HCl/ 1M NaCl/ 100mM NaH2PO4 pH 2.4/ 1% FA	
Quench buffer volume :	20 ul	20 ul
Number of peptides anayzed :	121	121
Sequence coverage :	99.0%	99.0%
Replicates :	3, 3, 3, 0, 0, 0	3, 3, 3, 0, 0, 0
Criteria for HDX rate difference :	Difference of HDX level at a given timepoint is > 8 % and > 1 Da and p values of student t-test is < 0.01	
Protein amount (pmol) :	78	78 + 116
