# Supplementary material for: Soluble αβ-tubulins reversibly sequester TTC5 to regulate tubulin mRNA decay
Source: Nat Commun. 2024 Nov 17;15:9963. doi: 10.1038/s41467-024-54036-0 (PMC11570694; doi:10.1038/s41467-024-54036-0)
Supplement: Supplementary file 3 — Description of Additional Supplementary Files [file 41467_2024_54036_MOESM3_ESM.pdf]

Description of Additional Supplementary Files

Supplementary Data 1: Analysis of TurboID-TTC5 TMT-MS data as plotted in Fig. 1g. A two-sided Student's t-test was used to calculate log2 fold-change differences and p-values between colchicine-treated samples (WT+Col) and untreated samples (WT-contr). No correction for multiple comparisons was applied.

Supplementary Data 2: Analysis of GFP-TTC5 label-free proteomics data as plotted in Fig. S1l. A two-sided Student's t-test was used to calculate log2 fold-change differences and p-values between colchicine-treated samples and control samples. No correction for multiple comparisons was applied.

Supplementary Data 3: Tables showing the differences in deuterium uptake between two states. For each state comparison, results are shown for both differences in deuterium uptake by percentage (%D) and by number of deuterons (#D). Largest differences are highlighted in different colors: blue indicates PROTECTION of the peptide and red indicates increased EXPOSURE (usually associated with allosteric modifications)
