# Supplementary material for: Soluble αβ-tubulins reversibly sequester TTC5 to regulate tubulin mRNA decay
Source: Nat Commun. 2024 Nov 17;15:9963. doi: 10.1038/s41467-024-54036-0 (PMC11570694; doi:10.1038/s41467-024-54036-0)
Supplement: Supplementary file 7 — Transparent Peer Review file [file 41467_2024_54036_MOESM7_ESM.pdf]

nature portfolio

Peer Review File

Soluble αβ-tubulins reversibly sequester TTC5 to regulate tubulin mRNA decay

Corresponding Author: Professor Ivana Gasic

Version 0:

Reviewer comments:

Reviewer #1

(Remarks to the Author)
The HDX results are clear and conclusion is convincing. The only concern is regarding the first time point selected for the HDX experiments. The authors took 3-seconds as the first time point for HDX MS. It is critical to allow enough mixing during HDX experiment for each time point so the results can be reproducible. Please illustrate how the authors manage to make this extremely short time point reproducible.

Reviewer #2

(Remarks to the Author)
In this study, Batiuk and colleagues discovered that soluble αβ-tubulins in the cytosol act as inhibitors of TTC5, thereby preventing TTC5-mediated tubulin mRNA decay. Initially, the authors used colchicine-treated cytosol fractions versus normal condition cytosol to perform TTC5 baited pull-down. They found that the αβ-tubulin band diminished in the treatment group. Subsequently, they tested the Kd value of αβ-tubulin interacting with TTC5 in vitro, demonstrating that this process can occur within cells. Following this, they discovered that colchicine treatment enables TTC5 to pull down mRNA of α-tubulin and β-tubulin. They used mass spectrometry (MS) to test the change in the amount of proteins interacting with TTC5. Proximity labeling was also conducted during this process. The authors then conducted Hydrogen-Deuterium Exchange Mass Spectrometry (HDX-MS) and found that the C-terminal of TTC5 can interact with both the αβ-tubulin and the nascent tubulins at the ribosome. They proposed a model in which the C-terminal of TTC5 acts as a switch between being inhibited by αβ-tubulin under normal conditions and binding to nascent tubulins to induce downstream degradation of the tubulin mRNAs after colchicine induction. They made some mutations that abolished the binding of TTC5, resulting in TTC5 becoming constitutively active. Finally, they conducted knockout (KO) experiments and introduced the mutations in mitotic cells, finding that the constitutively active TTC5 caused mitotic defects.

This research focuses on a very specific aspect of tubulin autoregulation and reveals a key factor regulating the activity of TTC5. It is significant in the field of tubulin regulation. However, as stated in the article, the research is incomplete due to the missing link between colchicine induction and the TTC5 switch. This does not diminish the value of the conclusions stated in the article, but it does somewhat weaken the logical chain in the biological process. In summary, I suggest that the manuscript might be more suitable for a specialized journal rather than a broad scientific audience like that of Nature Communications.

Major :
1. The entire experiment lacks evidence of the process occurring under normal conditions without treatment. If αβ-tubulin only dissociates from TTC5 upon colchicine treatment, then the discovery would be less meaningful. The association and dissociation need to be tested in normal cells during normal cellular activities related to microtubules, such as the cell cycle.
2. All of the αβ-tubulin bands in the western blotting need to be stained with antibodies of α-tubulin and β-tubulin respectively to show that the band is a mixture of these two types of tubulin monomers.
3. There is no direct evidence in this research that TTC5 directly binds either the dimer or the monomer. Western blotting and similar biochemical experiments can only prove that they can form a complex; AlphaFold prediction can only prove that they have the capacity for binding, but this may not be the case under physiological conditions. At least an experiment like GST-pull down needs to be added.
4. The experiment only tested the co-immunoprecipitation (co-IP) with exogenous recombinant TTC5. Endogenous TTC5 co-IP with its antibody or with a tag knocked in should be tested.

Minor:
1. Based on the datasets in FigS1.H and FigS1.C, it appears that TTC5 exhibits a differential preference between α-tubulin

and β-tubulin. The verification of this preference, as well as its cause and effect, warrant further investigation.
2. According to the datasets in FigS1.H and Fig1.G, tubulins are not among the highest fold change (FC) candidates, and some are barely significant. Therefore, these details need to be addressed, and candidates with better FC and p-value should be analyzed.
3. The structural model of TTC5 itself, as well as its interaction with tubulins, could be regenerated using AlphaFold3.
4. This experiment does not directly demonstrate TTC5 binding with nascent tubulin monomers. One suggestion is to knock in a tag at the N-terminal of α-tubulin and β-tubulin. The interaction of TTC5 with nascent tubulins could then be tested by co-IP.
5. In the BiFC experiment, a flow cytometry analysis showing the percentage of cells successfully expressing the exogenous protein should be included. This would rule out the possibility that any observed differences are due to transfection issues.
6. In Figure3.F, it would be beneficial to add a combination of α and β-tubulins to simultaneously verify the loss of capacity in binding the dimer.
7. In Figure3.F, a ribosomal protein exhibits stronger binding to TTC5(D175A) while other ribosomal protein bands diminish. This protein may be actively involved in this process and is worth further examination.
8. All bands annotated in the SYPRO Ruby staining should be removed, as there is no evidence to suggest that the band represents the protein without merging the western blotting band and the Ruby band.
9. In the proximity labeling experiments in FigS1.J/K and FigS2.C, a control group of TurboID+/Biotin- should be added to better reduce the background. Additionally, the blotting of TUBB should be co-tested with TUBA.
10. The proteins identified in the co-IP experiment differ from those found in the proximity labeling experiment. Please analyze the difference between the two lists. In addition to this, the proximity labeling experiment, which involves the KI of TurboID into the TTC5 gene, can be conducted in live cells. This ensures that the quantity and subcellular location of TurboID-TTC5 closely resemble those under normal conditions. It also helps to avoid the labeling of irrelevant proteins due to exogenous expression.

Reviewer #3

(Remarks to the Author)
Remarks to the authors:
Batiuk et al report that reversible sequestration of TTC5 via its interactions with soluble αβ-tubulin heterodimers serves as a key step in tubulin autoregulation, a process involving selective decay of tubulin-encoding mRNAs to secure tight control of the available αβ-tubulin. The authors identify the flexible C-terminal tail of CCT5 as a molecular switch, which, depending on conditions, stabilizes either repressive interactions of TTC5 with soluble αβ-tubulins or activating interactions with nascent tubulin on the ribosome. They further find that loss of sequestration by soluble αβ-tubulins constitutively activates TTC5, leading to reduced tubulin mRNA levels and causing mitotic defects.
This is a well-thought-out and meticulously executed study that sheds important new light on a critical missing piece of the long-standing enigma of tubulin autoregulation. The study also creates and leaves open several questions about tubulin autoregulation, which is understandable. I would like the authors to address the comments that I list below.

Major comments:
1) An intuitive thought would be that depolymerization of microtubules would increase, at least temporarily, the intracellular concentration of soluble αβ-tubulins, which, according to the proposed model and biochemical data, should increase sequestration of TTC5 and thus stabilize tubulin-encoding mRNAs. In contrast, the opposite is the case, tubulin depolymerization weakens the interaction with TTC5 due to, as the authors argue in the discussion, “some change” to αβ-tubulins. It would be helpful to the reader if the authors included in the introduction some comments on what is known and unknown about qualitative and quantitative changes to soluble αβ-tubulins when microtubules polymerize and depolymerize.

2) Related to the point above, based on the results, one might expect that enhanced polymerization of microtubules would lead to an acute depletion of soluble αβ-tubulins, loss of TTC5 sequestration, and diminished tubulin mRNA levels. However, this would jar with a common sense that increased demand for tubulins should favor stabilization of the encoding mRNAs. Have the authors considered experiments with inhibitors of microtubule depolymerization? I suggest that some of these experiments, e.g., looking at CCT5 activity and effect on tubulin mRNA levels upon treatment, be included in the manuscript to further probe the proposed model.

3) Colchicine is used for nearly all experiments where microtubule destabilization is considered, except for Fig. S1I where loss of TTC5-tubulin interactions is validated with nocodazole. It would be important to see if tubulin mRNA levels respond to treatment with nocodazole or other microtubule-destabilizing drugs similarly to colchicine treatment (Figs. 2G, S2D, 3H, S3I).

4) Could the authors elaborate on the reversibility of TTC5 sequestration? Fig. 1D shows that soluble porcine brain αβ-tubulins can outcompete (extract) CCT5 from its complex with RNCs. Is the opposite also the case, i.e., that increasing concentrations of RNCs progressively interrupt interactions of TTC5 with soluble αβ-tubulins? On that note, and with reference to the point 1 above, how do the authors envisage the progressively weaker association of soluble αβ-tubulins with TTC5 comes about upon microtubule destabilization? It would seem that whatever molecular event is responsible for the weaker TTC5/αβ-tubulin association would have to affect both pre-existing soluble αβ-tubulins as well as any newly depolymerized αβ-tubulin heterodimers to allow RNCs to gain competitive advantage for TTC5. Some discussion of this should be included in the text.

5) Has the interaction interface between TTC5 and αβ-tubulins been predicted by AlphaFold and, if so, did it confirm the significance of D175 in TTC5? How were the “various conserved surface residues” in TTC5 chosen for analysis by mutagenesis (several acidic residues were selected, but not from the TTC5 segments that showed differential deuteration by HDX-MS)? It is impressive that a binding-defective point mutant was identified.

6) I recommend that for predictions of interactions between TTC5 and nascent β-tubulin by AlphaFold the authors employ a more rigorous approach and show an alignment (and report a number) of converging predictions out of several independently generated structure predictions. For instance, see Bonneau et al, Genes Dev 2023 (PMID: 37399331) or Shah et al, Nat Commun 2024 (PMID: 38605040). A section on how the predictions were generated and fitted into the cryoEM density should be included in Methods. Are similar interactions also predicted to occur between the C-terminal tail of TTC5 and the N-terminal region of α-tubulin?

Minor Comments:
- Line 74: “…simply overexpressing TTC5 in cells is insufficient to trigger tubulin mRNA degradation (Figure S1C)…”. As I understand it, what was done here was really an add-back of WT CCT5 to TTC5 KO cells, i.e., rescue, not an overexpression sensu stricto. One might expect that if the level of transgenic TTC5 protein indeed highly exceeded the endogenous TTC5 level, this would suffice to trigger tubulin mRNA degradation (since much of TTC5 could not be sequestered due to the limited soluble αβ-tubulin). Please revise and include a western of TTC5 in WT, KO and rescue cells.

- Lines 87-89: “Because the cell lysates were prepared on ice, both the untreated and colchicine-treated cytosol had equal amounts of depolymerized αβ-tubulins, with little or no intact microtubules (whose polymerization is temperature-dependent).” Can the authors qualify what they mean by “depolymerized αβ-tubulins”? Are these strictly αβ heterodimers or potentially higher-order, partially polymerized tubulin structures? Does cell lysis lead to the same type of depolymerized αβ-tubulins as colchicine treatment? Please also cite a reference documenting temperature dependence.

- What does “crosslinking” refer to in the “nascent β-tubulin crosslinking assay” (Fig. 1D)?

- Line 107: “Colchicine-triggered loss of a TTC5 inhibitor”. I am not sure that inhibitor is the best word to use here. Please rephrase.

- Fig. 2H (lines 689 and 690): the MHQV mutant of the MREI motif is not discussed. Please include a citation and explain where this motif occurs in β-tubulin.

- Figs. 3B and S3B: two samples are labeled as WT; the one on the right is presumably KO, not WT?

- Lines 225, 226: “In agreement with previous reports…” – please cite these reports.

- Fig. S4A should be cited before S4B.

- What are the data in Fig. 1F normalized against?

- Figs. 2H and 3F: what does “TUB 60+4 aa” indicate? Also, no radioactive labeling is mentioned, but autoradiography was employed – this should be explained in the figure legend and in Methods.

- It would be good to demonstrate in Fig. S1A that the faint upper band is indeed TTC5, although this is later shown in Fig. 3B.

- What is PAR is Fig. S4B?

Version 1:

Reviewer comments:

Reviewer #1

(Remarks to the Author)
The authors have addressed my concerns.

Reviewer #2

(Remarks to the Author)
I would like to thank the authors for addressing my comments. I am satisfied with their responses in general.

Reviewer #3

(Remarks to the Author)
The authors have satisfactorily addressed all points that I raised. I have no further comments.

Open Access This Peer Review File is licensed under a Creative Commons Attribution 4.0 International License, which permits use, sharing, adaptation, distribution and reproduction in any medium or format, as long as you give appropriate credit to the original author(s) and the source, provide a link to the Creative Commons license, and indicate if changes were made.
In cases where reviewers are anonymous, credit should be given to 'Anonymous Referee' and the source.
The images or other third party material in this Peer Review File are included in the article’s Creative Commons license, unless indicated otherwise in a credit line to the material. If material is not included in the article’s Creative Commons license and your intended use is not permitted by statutory regulation or exceeds the permitted use, you will need to obtain permission directly from the copyright holder.
To view a copy of this license, visit https://creativecommons.org/licenses/by/4.0/

Point-by-point response to reviewer comments:

Reviewer #1 (Remarks to the Author):

The HDX results are clear and conclusion is convincing. The only concern is regarding the first time point selected for the HDX experiments. The authors took 3-seconds as the first time point for HDX MS. It is critical to allow enough mixing during HDX experiment for each time point so the results can be reproducible. Please illustrate how the authors manage to make this extremely short time point reproducible.

As now stated in the Methods, 3 seconds is estimated by counting the up-and-down-pipetting cycles in lieu of a timer. Reproducibility is due to the experimentalist's experience. The standard deviation for the 3-second timepoint is comparable to other time points (between 1% and 2.3%).

Line 614-615:

"The 3 seconds deuteration is estimated by counting the up-and-down-pipetting cycles in lieu of a timer."

Reviewer #2 (Remarks to the Author)

Major :

1. The entire experiment lacks evidence of the process occurring under normal conditions without treatment. If αβ-tubulin only dissociates from TTC5 upon colchicine treatment, then the discovery would be less meaningful. The association and dissociation need to be tested in normal cells during normal cellular activities related to microtubules, such as the cell cycle.

We agree it is important to establish the physiologic relevance of our key discovery that αβ-tubulin is a sequestration factor for TTC5. We apologize that we did not properly emphasize how physiologic relevance was established in our paper, leaving the reader with a mistaken impression that our findings only relate to artificial conditions. As the referee will know, a classic strategy of molecular genetics to establish the physiologic role of a putative interaction is to identify a mutation that selectively disrupts the interaction and investigate the consequences of that perturbation. This is precisely what we did: two independent mutants that selectively disrupt the tubulin-TTC5 interaction (Fig. 3B) were introduced into cells lacking endogenous TTC5 and shown to constitutively degrade tubulin mRNA (Fig. 3G) and cause aberrant chromosome segregation (a microtubule-dependent process) during mitosis (Fig. 4B, 4C). Importantly, neither the constitutive degradation of tubulin mRNA nor the chromosome segregation defects employ colchicine or other treatments, and examine the consequences under normal conditions as requested by this referee.

A second point of potential confusion that we apparently did not explain sufficiently clearly is that colchicine <u>does not</u> dissociate TTC5 from αβ-tubulin. Indeed, the *in vitro* sequestration and inhibition of TTC5 by αβ-tubulin is impervious to colchicine (Fig. 1E, lane 2; Fig. S1H). Instead, it is a downstream <u>consequence</u> of microtubule disruption in living cells that triggers TTC5 dissociation. This is analogous to many other stress responses. For example, treatment of cells with DTT triggers phosphorylation of eIF2α in cells, but DTT has no effect on that phosphorylation reaction *in vitro*. Rather, it is the signaling pathway activated by DTT (i.e., the UPR) that leads to the downstream consequences, making DTT a very useful tool in probing this signaling pathway. In the same way, we are using colchicine (and other microtubule perturbing agents) as a convenient tool to activate the tubulin autoregulation pathway, then using the information gained from this analysis to probe the pathway's relevance in non-artificial situations as outlined above.

With the above explanations (now incorporated into the revised text), a reader can better appreciate the biological relevance of our findings under non-colchicine conditions, and also recognize that colchicine is not artifactually dissociating TTC5. We also note that tubulin autoregulation is established as a biologically important process in cells and in humans via analysis of mutants that selectively perturb different factors in the pathway (see Höpfler et al., 2023, and references therein).

Line 245-247:

"To investigate the physiological importance of αβ-tubulin-mediated repression of TTC5 in conditions that do not require chemical perturbation of the microtubule network we leveraged the constitutively active TTC5 mutants, $TTC5^{D175A}$ and $TTC5^{T432E}$, and assessed mitotic fidelity in living cells using microscopy (Figure 4A)."

Line 113-118:

"Thus, colchicine treatment of live cells, but not cytosol or purified tubulins, triggers a yet-unidentified signal that changes the capacity of soluble αβ-tubulins to interact with TTC5. This loss of interaction with αβ-

tubulins is a downstream consequence of microtubule disruption and correlates with the disappearance of a TTC5-inhibitory activity in cytosol as monitored using in vitro assays, and further correlates with the initiation of tubulin mRNA decay in cells[9].”

2. All of the αβ-tubulin bands in the western blotting need to be stained with antibodies of α-tubulin and β-tubulin respectively to show that the band is a mixture of these two types of tubulin monomers.

The mass spectrometry data (Fig. 1G and S1L) establish that TTC5 interacts with both the α- and β-tubulin subunits. To highlight this, we now color-code the two isoforms separately in the plots (Fig. 1G and S1L) and blot for both subunits in key validation experiments (Fig. S1E and S1F). Finally, we emphasize that the αβ-tubulin heterodimer is exceptionally stable and that individual subunits are not observed under physiological conditions (Caplow and Fee, MBC, 2002, PMID: 12058074). The only known exception is during initial synthesis and assembly of the αβ-heterodimer, when the individual subunits are protected by tubulin-specific chaperones (Gestaut et al, Cell, 2022, PMID: 36493755; Kelly et al, NSMB, 2022, PMID: 35449234). As these chaperones are not hits in our TTC5 interactome, we can be confident that TTC5 interacts with the αβ-heterodimer, allowing us to use one subunit as a proxy for the heterodimer.

3. There is no direct evidence in this research that TTC5 directly binds either the dimer or the monomer. Western blotting and similar biochemical experiments can only prove that they can form a complex; AlphaFold prediction can only prove that they have the capacity for binding, but this may not be the case under physiological conditions. At least an experiment like GST-pull down needs to be added.

A pulldown experiment demonstrating that recombinant purified Strep-tagged TTC5 directly interacts with purified brain αβ-tubulin heterodimers is presented in Fig. S1H. This shows that TTC5 interacts directly with αβ-tubulin without a need for any other factors. To further strengthen this point, we have now included a western blot alongside a total protein stain from an immunoprecipitation experiment demonstrating direct binding of recombinant Strep-tagged TTC5 and purified brain αβ-tubulin heterodimers (Fig. S1F).

4. The experiment only tested the co-immunoprecipitation (co-IP) with exogenous recombinant TTC5. Endogenous TTC5 co-IP with its antibody or with a tag knocked in should be tested.

We agree that demonstrating an interaction at endogenous expression levels is important to exclude potentially promiscuous co-IP due to overexpression. To address this, we have exploited the fact that low-level leaky expression from the Dox-inducible promoter results in expression of tagged TTC5 at levels very close to endogenous levels (see blots in Fig. S1O). A proximity-labelling experiment performed under these conditions verifies that at near-endogenous levels TurboID-tagged TTC5 selectively biotinylates αβ-tubulins preferentially before but not after colchicine treatment. (Fig. 1G, S1O, S1P). We opted to do this experiment using proximity labelling rather than pull down because it allowed us to test more directly the binding of TTC5 to αβ-tubulins in living cells at near-endogenous levels.

<u>Minor:</u>
1. Based on the datasets in FigS1.H and FigS1.C, it appears that TTC5 exhibits a differential preference between α-tubulin and β-tubulin. The verification of this preference, as well as its cause and effect, warrant further investigation.

Any minor differences between α- and β-tubulins in Fig. S1H and S1C (now S1L and S1D) are not statistically significant, which is why we have not investigated this further. In all experiments of the tubulin autoregulation pathway, we have not seen any appreciable difference in α- and β-tubulin mRNAs as targets of TTC5-mediated decay (Fig. 2G, 3G, 3H; also Gasic et al, Plos Biol, 2019, PMID: PMID: 30964857; Lin et al, Science, 2020, PMID: 31727855, Höpfler et al, MolCel, 2023, PMID: 37295431), and have not seen differences in the physical interaction between TTC5 and α- and β-tubulin protein (Fig. 1G, 2H, 3F, S1L) which is one reason we conclude that TTC5 engages the αβ-tubulin heterodimer. In support of this conclusion, we now provide a high-resolution SDS-PAGE separation alongside a western blot of the tubulin subunits in key co-immunoprecipitation experiments (Fig. S1E and S1F), showing a stoichiometric ratio of the TTC5-coimmunoprecipitated α- and β-tubulin subunits (see SYPRO Ruby stained gels in Fig. S1E and S1F). Thus, we do not feel there is anything actionable regarding a potential preference for one subunit versus the other.

2. According to the datasets in FigS1.H and Fig1.G, tubulins are not among the highest fold change

(FC) candidates, and some are barely significant. Therefore, these details need to be addressed, and candidates with better FC and p-value should be analyzed.

Fold-change is not the sole criterion by which candidates for a biologically relevant sequestration factor were chosen. Plausible candidates must also be conserved, located in the correct cellular compartment, expressed in all cell types and tissues, of sufficient abundance to act as a sequestration factor, and physically interact with TTC5 with sufficient stability to realistically sequester it. When all of these criteria are applied to the results, only the tubulins emerge as candidates. For example, in Fig. 1B, there is only one component that is both abundant enough and associated tightly enough to be a realistic sequestration factor. Thus, our choice was not arbitrary but based on the available knowledge of the tubulin autoregulation pathway. We now clarify this in the revised paper.

Line 130-132:

"Manual annotation based on the conservation, subcellular location, ubiquitous expression, and abundance, revealed tubulins as the only possible TTC5 sequestration factors."

3. The structural model of TTC5 itself, as well as its interaction with tubulins, could be regenerated using AlphaFold3.

We used both AF2 and AF3 to predict structures of α- and β-tubulin bound TTC5, but neither produced high-confidence complexes. We therefore do not use these predictions in our paper. There are experimental structures of the individual components (TTC5 and the tubulins), and the AF2 and AF3 predictions match these very well.

4. This experiment does not directly demonstrate TTC5 binding with nascent tubulin monomers. One suggestion is to knock in a tag at the N-terminal of α-tubulin and β-tubulin. The interaction of TTC5 with nascent tubulins could then be tested by co-IP.

Rigorous evidence for TTC5 engaging nascent tubulin monomers is provided in earlier work (Lin et al., 2019, Science, PMID: 31727855; Höpfler et al., 2023, Mol. Cell, PMID 37295431). This evidence includes site-specific UV-activated photocrosslinking studies, a cryo-EM structure of the TTC5-ribosome complex, and substantial structure-guided mutagenesis. Because adding even a single amino acid to the N-terminus of nascent tubulin disrupts its recognition by TTC5 (Lin et al, 2019, Science, PMID: 31727855), N-terminal tags are unsuitable for such analyses.

5. In the BiFC experiment, a flow cytometry analysis showing the percentage of cells successfully expressing the exogenous protein should be included. This would rule out the possibility that any observed differences are due to transfection issues.

The BiFC constructs are stably expressed from one open reading frame and under a doxycycline-inducible promoter, ensuring equal levels of expression of both TTC5 and the tubulin counterpart in the same cells (now included as a new Fig. S3F). We initially identified cells expressing the constructs (based on Venus fluorescence), then analyzed only those cells. This strategy excludes any issues due to transfection differences. We have now clarified this in the Materials and Methods.

Line 490-496:

"Image segmentation was performed using a custom module editor MetaXpress from Molecular Devices. Masks were generated to extract Venus fluorescence intensity across all conditions. Briefly, cell nuclei and body masks were created using SiR-DNA to define a master object (all cells). An automated data analysis pipeline was then applied to identify cells that either express or do not express the constructs based on Venus's fluorescence intensity. Quantitative analysis was carried out only on cells with detectable fluorescence intensity and values were plotted using GraphPad Prism 8."

6. In Figure3.F, it would be beneficial to add a combination of α and β-tubulins to simultaneously verify the loss of capacity in binding the dimer.

The loss of capacity of the mutant TTC5 proteins in binding the tubulin αβ-heterodimer in a pulldown experiment is already shown in Fig. 3B.

7. In Figure3.F, a ribosomal protein exhibits stronger binding to TTC5(D175A) while other ribosomal protein bands diminish. This protein may be actively involved in this process and is worth further examination.

The protein noted by the referee is not a ribosomal protein, as it is present in the TTC5(D175A) protein prep (now demonstrated in new Fig. S3H), which seems to have a bit more degradation products. This explains why it is also observed in the sample where the TTC5-binding motif of nascent tubulin is mutated (lane 5 in Fig. 3F). This is why we did not pursue this further.

8. All bands annotated in the SYPRO Ruby staining should be removed, as there is no evidence to suggest that the band represents the protein without merging the western blotting band and the Ruby band.

The only products that have been annotated are Strep-TTC5 and ribosomal proteins. We are confident in both assignments based on experiments where the indicated factor was omitted, and the respective band(s) disappeared. For example, omission of Strep-TTC5 in lane 1 (Fig. 3F) causes that band to disappear. Mutation of the nascent chain to prevent TTC5-ribosome binding (rigorously established in Lin et al., Science, 2019) causes the ribosomal proteins to disappear (lane 2 in Fig. 3F). Other bands whose identities are not established are not labelled. To support this claim, we now include western blot data in the key experiment (Fig. 3F).

9. In the proximity labeling experiments in FigS1.J/K and FigS2.C, a control group of TurboID+/Biotin- should be added to better reduce the background. Additionally, the blotting of TUBB should be co-tested with TUBA.

The Biotin(+)/TurboID-TTC5(-) condition is the appropriate control to identify proteins are specific interactors of TurboID-TTC5. Omitting biotin is not helpful because there is already biotin in the culture medium, serum, and cells (as it is an essential vitamin). Although one could use biotin starvation to deplete it from cells, this impairs cell homeostasis and growth, leading to potential artefacts. As explained above, α- and β-tubulins form constitutive heterodimers in cells, so most experiments only blot for one as a proxy for the heterodimer.

10. The proteins identified in the co-IP experiment differ from those found in the proximity labeling experiment. Please analyze the difference between the two lists. In addition to this, the proximity labeling experiment, which involves the KI of TurboID into the TTC5 gene, can be conducted in live cells. This ensures that the quantity and subcellular location of TurboID-TTC5 closely resemble those under normal conditions. It also helps to avoid the labeling of irrelevant proteins due to exogenous expression.

The proximity labelling experiments were performed in live cells with TurboID-TTC5 expressed at near-endogenous levels (as now documented in Fig. S1O). Co-IP and proximity labelling necessarily identify different proteins because they are probing different parameters. Co-IP identifies proteins whose interaction (either direct or indirect) is sufficiently strong and stable to survive substantial dilution and lengthy biochemical procedures. By contrast, proximity-labelling identifies proteins containing exposed lysine residues within a radius of ~10 nm (Dae In Kim et al., 2014, PNAS, PMID: 24927568) in the native cellular environment. Both methods are prone to background (albeit for qualitatively different reasons), which is why shared hits are far more likely to be strong and direct interaction partners. Our study sought to identify a sequestration factor, which necessarily needs to be a direct and stable interactor. This is why we focused on the shared hits, and why we felt highlighting or pursuing the differences was a distraction (although the full datasets are made available for further analysis by the community).

Reviewer #3 (Remarks to the Author):

Major comments:

1) An intuitive thought would be that depolymerization of microtubules would increase, at least temporarily, the intracellular concentration of soluble αβ-tubulins, which, according to the proposed model and biochemical data, should increase sequestration of TTC5 and thus stabilize tubulin-encoding mRNAs. In contrast, the opposite is the case, tubulin depolymerization weakens the interaction with TTC5 due to, as the authors argue in the discussion, "some change" to αβ-

tubulins. It would be helpful to the reader if the authors included in the introduction some comments on what is known and unknown about qualitative and quantitative changes to soluble αβ-tubulins when microtubules polymerize and depolymerize.

We thank the reviewer for this suggestion and have now included in the introduction what is known about the qualitative and quantitative changes in αβ-tubulins upon microtubule depolymerization.

Line 53-59:

"Although the precise signal generated by the rise in soluble αβ-tubulins that triggers tubulin autoregulation is yet to be identified, both qualitative and quantitative changes are known to occur in soluble αβ-tubulins. These changes include posttranslational modifications, such as tyrosination and ubiquitination, and interactions with specific binding partners, such as stathmin and CLIP-170[5,19]. Additionally, depolymerization presumably renders αβ-tubulins accessible to degradation machineries[20,21]."

2) Related to the point above, based on the results, one might expect that enhanced polymerization of microtubules would lead to an acute depletion of soluble αβ-tubulins, loss of TTC5 sequestration, and diminished tubulin mRNA levels. However, this would jar with a common sense that increased demand for tubulins should favor stabilization of the encoding mRNAs. Have the authors considered experiments with inhibitors of microtubule depolymerization? I suggest that some of these experiments, e.g., looking at CCT5 activity and effect on tubulin mRNA levels upon treatment, be included in the manuscript to further probe the proposed model.

We and others have done such experiments and find driving microtubule assembly with taxol stabilizes tubulin mRNAs (new Fig. S1N; Cleveland et al, Cell, 1981, PMID: 6116546; Gasic et al, Plos Biol, 2019, PMID: 30964857). Now included in the manuscript are data showing that treatment with microtubule-stabilizing drugs does not cause TTC5 to dissociate from αβ-tubulins (Fig. S1M). We recognise this is counterintuitive, and the reason is nuanced as follows. TTC5 is present in cells at 35 nM, whereas the concentration of αβ-tubulins is around 10 µM (with about 30% being free in normal cultured cells; Hiller and Weber, Cell, 1978, PMID: 688394; Zhai and Borisy, JCS, 1994, PMID: 8056844). Even with microtubule stabilisation by taxol, the concentration of free αβ-tubulins is still 1 µM or more. Thus, free tubulin always greatly exceeds TTC5, meaning that free tubulin concentration is not directly controlling TTC5 sequestration. Instead, a change in the free:polymer ratio of tubulins initiates a signal that indirectly modulates the tubulin-TTC5 interaction. We arrived at this conclusion because an identical amount of unpolymerized αβ-tubulin from untreated versus colchicine-pretreated cells has markedly different TTC5-sequestration activity. Furthermore, even though colchicine acts within seconds to depolymerize microtubules, the effects on TTC5-sequestration begin after ~15 min, indicating a yet-to-be-identified signal that takes time to influence the tubulin-TTC5 interaction. Thus, the pathway is complex and multifaceted, not unlike many other stress responses that have been characterized.

3) Colchicine is used for nearly all experiments where microtubule destabilization is considered, except for Fig. S1I where loss of TTC5-tubulin interactions is validated with nocodazole. It would be important to see if tubulin mRNA levels respond to treatment with nocodazole or other microtubule-destabilizing drugs similarly to colchicine treatment (Figs. 2G, S2D, 3H, S3I).

We and others have established that tubulin mRNA levels respond to a wide range of microtubule depolymerizing drugs (such as colchicine, nocodazole, or combretastatin A4; Cleveland et al, 1981, Cell, PMID: 6116546; Lin et al, 2019, Science, PMID: 31727855; Höpfler et al., 2023, Mol. Cell, PMID 37295431) as well as various physiological and toxic inputs (Gasic et al, 2019, PloS Biol, PMID: 30964857). This key point is now mentioned in the paper.

Line 50-53:

"In cells, various physiological and toxic stimuli can destabilize microtubules, leading to an increase in the proportion of soluble αβ-tubulins and initiating tubulin autoregulation[16,17]. Experimentally, this can be replicated using several microtubule-depolymerizing agents, such as colchicine, nocodazole, or combretastatin A4[9,14,15,18]."

4) Could the authors elaborate on the reversibility of TTC5 sequestration? Fig. 1D shows that soluble porcine brain αβ-tubulins can outcompete (extract) CCT5 from its complex with RNCs. Is the opposite also the case, i.e., that increasing concentrations of RNCs progressively interrupt interactions of TTC5 with soluble αβ-tubulins? On that note, and with reference to the point 1 above, how do the authors envisage the progressively weaker association of soluble αβ-tubulins with TTC5 comes about upon microtubule destabilization? It would seem that whatever molecular event is responsible for the weaker TTC5/αβ-tubulin association would have to affect both pre-

existing soluble αβ-tubulins as well as any newly depolymerized αβ-tubulin heterodimers to allow RNCs to gain competitive advantage for TTC5. Some discussion of this should be included in the text.

The competition experiment with nascent tubulin-displaying RNCs cannot be performed because they cannot be produced in anywhere near the concentrations needed. Nonetheless, our structural insights suggest that TTC5 binding to αβ-tubulins and nascent tubulin RNCs is mutually exclusive. This is because TTC5's C-terminal tail, which is needed for αβ-tubulin interaction, is needed for and sequestered by nascent tubulin. The sequence of events from microtubule depolymerization to a weakened TTC5-αβ-tubulin complex is unknown, but is now discussed in the paper.

Line 286-290:

"Such posttranslational modifications would have to be reversible and occur on both the pre-existing and newly depolymerized tubulins to allow tubulin-translating ribosomes to gain competitive advantage for TTC5. The observed 15-60-minute timeframe required for TTC5 to translocate from αβ-tubulins to tubulin-translating ribosomes (Figure 1E-F) may reflect the time required to posttranslationally modify a large pool of unpolymerized αβ-tubulins."

5) Has the interaction interface between TTC5 and αβ-tubulins been predicted by AlphaFold and, if so, did it confirm the significance of D175 in TTC5? How were the "various conserved surface residues" in TTC5 chosen for analysis by mutagenesis (several acidic residues were selected, but not from the TTC5 segments that showed differential deuteration by HDX-MS)? It is impressive that a binding-defective point mutant was identified.

We used both AF2 and AF3 to predict structures of α- and β-tubulin bound TTC5, but neither produces high-confidence complexes. We therefore do not use these predictions in our paper. Conserved surfaces were identified using ConSurf, a tool that maps conservation onto the experimental structures of TTC5 (see Höpfler et al., 2023, Mol Cell, PMID: 37295431). This is now explained in the revised manuscript.

Line 207-210:

"In a second approach, we deployed ConSurf, a tool that maps conservation onto the experimental structures of proteins. This analysis identified various conserved surface residues in TTC5 (Figure S3A), which we mutated and found by pulldown that TTC5D175A was markedly reduced in its ability to bind αβ-tubulins (Figures 3B, S3B).“

6) I recommend that for predictions of interactions between TTC5 and nascent β-tubulin by AlphaFold the authors employ a more rigorous approach and show an alignment (and report a number) of converging predictions out of several independently generated structure predictions. For instance, see Bonneau et al, Genes Dev 2023 (PMID: 37399331) or Shah et al, Nat Commun 2024 (PMID: 38605040). A section on how the predictions were generated and fitted into the cryoEM density should be included in Methods. Are similar interactions also predicted to occur between the C-terminal tail of TTC5 and the N-terminal region of α-tubulin?

We now include a supplemental figure showing that the top ten AlphaFold2 multimer predictions of TTC5 with the N-terminal peptides of both α- and β-tubulins are high-confidence and nearly identical (Fig. S2E-F). The methods used for predicting the AlphaFold models and fitting of the model of TTC5 with the β-tubulin nascent chain into the cryo-EM density map have been described in an earlier paper, and reanalyzed here (8BPO, Höpfler et al, 2023, Mol Cell, PMID: 37295431).

Minor Comments:

- Line 74: "…simply overexpressing TTC5 in cells is insufficient to trigger tubulin mRNA degradation (Figure S1C)…". As I understand it, what was done here was really an add-back of WT CCT5 to TTC5 KO cells, i.e., rescue, not an overexpression sensu stricto. One might expect that if the level of transgenic TTC5 protein indeed highly exceeded the endogenous TTC5 level, this would suffice to trigger tubulin mRNA degradation (since much of TTC5 could not be sequestered due to the limited soluble αβ-tubulin). Please revise and include a western of TTC5 in WT, KO and rescue cells.

We now provide a western blot showing the expression levels of TTC5 across the relevant cell lines (Fig. S1C). The various TTC5 constructs were indeed reintroduced in TTC5 knockout cells under a doxycycline-inducible promoter. Doxycycline-induced expression of the transgenes results in an overexpression

compared to parental cell lines. However, since endogenous TTC5 is estimated to be only ~35 nM in cells, even a 100-fold overexpression can potentially be buffered by soluble αβ-tubulins (estimated to be in the low µM range). Furthermore, TTC5 alone is not sufficient to trigger tubulin mRNA decay; SCAPER and CCR4-NOT are also required, and these might also be under regulatory control.

- Lines 87-89: "Because the cell lysates were prepared on ice, both the untreated and colchicine-treated cytosol had equal amounts of depolymerized αβ-tubulins, with little or no intact microtubules (whose polymerization is temperature-dependent)." Can the authors qualify what they mean by "depolymerized αβ-tubulins"? Are these strictly αβ heterodimers or potentially higher-order, partially polymerized tubulin structures? Does cell lysis lead to the same type of depolymerized αβ-tubulins as colchicine treatment? Please also cite a reference documenting temperature dependence.

We have now clarified this by including the term *"depolymerized αβ-tubulin heterodimers"* (line 98) and references on microtubule cold-sensitivity (line 99). Exposure to cold, rather than cell lysis itself, depolymerizes microtubules down to tubulin heterodimers, similar to the effects of colchicine, nocodazole, or combretastatin A4. None of these treatments are known to cause tubulin oligomerization. Note also that colchicine addition after cell lysis is insufficient to abolish its sequestration activity (Fig. S1H).

- What does "crosslinking" refer to in the "nascent β-tubulin crosslinking assay" (Fig. 1D)?

We have now clarified in the figure legend that the crosslinking assay refers to UV-induced photocrosslinking of the nascent chain, which has p-benzoyl-L-phenylalanine (Bpa) incorporated close to the TTC5 binding site, as previously described (Lin et al., 2019, Science, PMID: 31727855).

Line 717-724:

"Nascent β-tubulin crosslinking assay in the presence of the indicated concentrations of soluble porcine brain αβ-tubulins. The TTC5 crosslink is indicated and verified by immunoprecipitation in the bottom panel. 94-residue β-tubulin nascent chain was produced in rabbit reticulocyte lysate in the presence of ^{35}S-methionine and the UV-activated cross-linking amino acid p-benzoyl-L-phenylalanine (Bpa) was incorporated at position 7 by amber suppression. Ribosome-nascent chain complexes were isolated and incubated with porcine brain tubulin as indicated and cross-linked under UV light. Reactions were then immuno-precipitated with anti-TTC5 antibody or directly analyzed by SDS-PAGE and autoradiography."

- Line 107: "Colchicine-triggered loss of a TTC5 inhibitor". I am not sure that inhibitor is the best word to use here. Please rephrase.

We have now rephrased *"inhibitor"* to *"sequestration factor"* (line 119).

- Fig. 2H (lines 689 and 690): the MHQV mutant of the MREI motif is not discussed. Please include a citation and explain where this motif occurs in β-tubulin.

We have now clarified in the figure legend that the MREI-to-MHQV mutant refers to the first four amino acids of the β-tubulin nascent chain. We also reference the work demonstrating that the MHQV mutation abolishes TTC5 binding (Lin et al., 2019, Science, PMID: 31727855).

Line 763-764:

"β indicates a β-tubulin construct in which its TTC5-interacting N-terminal MREI motif has been mutated to autoregulation-incompatible MHQV[14]."*

- Figs. 3B and S3B: two samples are labeled as WT; the one on the right is presumably KO, not WT?

In both figures, there are two WT samples. One sample's expression is induced with doxycycline, while the other is not induced and serves as a control for nonspecific binding in the immunoprecipitation experiment. The addition of doxycycline is annotated under the western blots.

- Lines 225, 226: "In agreement with previous reports…" – please cite these reports.

We have now included the missing references (line 248).

- Fig. S4A should be cited before S4B.

We have now matched the order of citations with the figure.

- What are the data in Fig. 1F normalized against?

Normalization is relative to a ribosomal transcript (RPLP1), as now stated in the legend (line 730).

- Figs. 2H and 3F: what does "TUB 60+4 aa" indicate? Also, no radioactive labeling is mentioned, but autoradiography was employed – this should be explained in the figure legend and in Methods.

"TUB 60 + 4 aa" refers to the specific construct used for in vitro translation of tubulins (as previously published in Lin et al, 2019, Science, PMID: 31727855). This and the labelling are now mentioned, and for clarity updated to *"TUB NC"*.

- It would be good to demonstrate in Fig. S1A that the faint upper band is indeed TTC5, although this is later shown in Fig. 3B.

The identity of this band is now validated using a TTC5 KO cell line now included in Fig. S1A.

- What is PAR is Fig. S4B?

"PAR", now replaced with *"parental"*, refers to parental cell line.
